# Effect of Traffic Exposure on Sick Building Syndrome Symptoms among Parents/Grandparents of Preschool Children in Beijing, China

**DOI:** 10.1371/journal.pone.0128767

**Published:** 2015-06-18

**Authors:** Linyan Li, Gary Adamkiewicz, Yinping Zhang, John D. Spengler, Fang Qu, Jan Sundell

**Affiliations:** 1 Department of Environmental Health, Harvard School of Public Health, Boston, MA, United States of America; 2 Department of Building Science, Tsinghua University, Beijing, China; School of Public Health, Zhejiang University, CHINA

## Abstract

**Introduction:**

Sick building syndrome (SBS) includes general, mucosal and skin symptoms. It is typically associated with an individual's place of work or residence. The aim of this study was to explore the effect of traffic exposure on SBS symptoms in Beijing, China.

**Methods:**

From January to May, 2011, recruitment occurred at kindergartens in 11 districts in Beijing. Self-administered questionnaires were distributed by teachers to legal guardians of children and then returned to teachers. The questionnaire asked them to recall the presence of 12 SBS symptoms from the previous three months. Living near a highway or main road (within 200 meters) was used as a proxy for traffic exposure. Multivariable logistic regression was used to test the association between traffic exposure and a higher number of SBS symptoms, controlling for key covariates.

**Results:**

There were 5487 valid questionnaires (65.0% response rate). Univariate analysis showed that living near a main road or highway (OR = 1.40), female gender (OR = 1.44), and environmental tobacco smoking (ETS) (OR = 1.13) were significant risk factors for general symptoms. Grandparent’s generation (OR = 0.32) and home ownership (owner vs. renter) (OR = 0.89) were significant protective factors. The adjusted odds ratio (aOR) for the association between living close to a highway and general symptoms remained significant in the multivariable model (aOR = 1.39; 95% CI = 1.21: 1.59). ORs and aORs were similar for mucosal and skin symptoms.

**Conclusions:**

This study found traffic exposure to be significantly associated with SBS symptoms. This finding is consistent with current literature that indicates an association between adverse health effects and living near highway or main road.

## Introduction

Sick Building Syndrome (SBS) includes ailments which have generally been associated with an individual's place of work (office building) or residence[1]. SBS includes several types of symptoms, namely general symptoms (headache, fatigue, feeling heavy-headed and difficulty concentrating), mucous symptoms (eye, throat and nose irritations or coughing) and dermal symptoms (face, hands or scalp)[2]. The rapid increase of SBS in China has become a focus of public health interest in recent years [3–5]. This increase occurred over a short time period, implicating the role of environmental changes as compared to genetic changes [4].

There are a number of risk factors for SBS found in previous studies. Gender, atopy, psychosocial factors, and proximity to photocopiers have been reported to predict the number of symptoms [1, 6, 7]. Environmental tobacco smoking (ETS) is investigated in most studies but only in some was ETS found to be a significant risk factor for SBS symptoms [8]. SBS symptoms are less likely if the resident owns the building [9]. Signs of high air humidity in dwellings have been related to an increase of SBS symptoms [10, 11], and dampness and molds have been associated with increased incidence and decreased remission of SBS [12]. More commonly a “sensation of dryness” is a risk factor for SBS symptoms [13]. A low ventilation rate was also an important risk factor reported by several studies [13–17].

Since traffic related public health issues have raised large public health concern recently, a number of studies have explored the association between some individual symptoms and traffic, although few of them directly linked traffic exposure to SBS symptoms [18–20]. Living near heavy traffic contributes to indoor air pollution and exposures, which are associated with adverse respiratory or cardiovascular effects [18–23]. Association between pollutants generated by traffic and increased morbidity or mortality are strengthened when multiple pollution sources are examined [24–26]. Noise created by traffic may also cause psychological disturbance, hypertension and cardiovascular disease [27–30].

Several studies have characterized the spatial patterns of traffic related exposures [31–36], and demonstrated that pollutant levels can approach background concentrations at a distance of 200 meters [37]. Therefore, distance to a roadway can be used as a surrogate for exposures to traffic-based air pollution and noise. Although it is a crude measure compared to detailed validation models, it is frequently used [34–36] because it can be easily obtained through a questionnaire [38].

Though SBS has been widely studied in Denmark [39–41], Sweden [6, 9, 10, 14], Singapore [42–44] and the United States [45–47], few studies have been conducted in China. Furthermore, China’s traffic pollution is keeping pace with rapid urbanization [48], so it is meaningful and necessary to investigate the impact of traffic-related exposure on SBS symptoms. The aim of this paper is to study the association between traffic exposure and increased SBS symptoms controlling for other independent key household covariates.

## Methods

### Study population

The China, Children, Homes and Health (CCHH) study is a cross-sectional study of children’s health in China[3, 49, 50]. From January through May, 2011, kindergartens in 11 districts in Beijing (Dongcheng, Xicheng, Chaoyang, Fengtai, Haidian and Shijingshan, Tongzhou, Changping and Daxing, Mentougou and Fangshan) were invited to participate in the study. Questionnaires were distributed by teachers to the legal guardians of children and subsequently returned to teachers. An overview of the study, consent language, and details about privacy and participating in the survey were included at the beginning of the each survey, and by filling out the questionnaire participants consented to participation in the study. The guardians answered questions regarding the children’s and their own health conditions and characteristics of their home environments.

### Assessment of outcome

Outcomes were assessed by asking: During the last three months, have you had any (or more) of the following symptoms:

(1) Fatigue; (2) Feeling heavy headed; (3) Headache; (4) Nausea/Dizziness; (5) Stuffy or runny nose; (8) Hoarse, dry throat; (9) Cough; (10) Dry or flushed facial skin; (11) Scaling/ itching scalp or ears; (12) Hands dry, itching, red skin. For each symptom, there were three possible responses: (A) Always (every week); (B) sometimes; (C) Never.

Symptoms (1)-(5), symptoms (6)-(9) and symptoms (10)-(12) were grouped as general symptoms, mucous symptoms and dermal symptoms, respectively.[3] Always, sometimes and never were given score of 2, 1 and 0, respectively.[51] Symptom scores (SCs), ranging from 0 to 10, 0 to 8 and 0 to 6, were constructed by summing up the number of symptoms multiplied by the frequency score for each subject. Then, each symptom in the model was dichotomized as high and low, based on the median values in the population.

### Exposure assessment

Living near a highway or main road was used as a metric for traffic exposure. Each participant was asked: “Is your home near a highway or main road within 200 meters (yes/no)?” [32–36].

### Covariates

Important personal characteristics and environmental characteristics were included in the model [8, 10, 38], including: (1) gender (female/male); (2) generation (parent/grandparent); (3) home ownership (own/rent); (4) Environmental Tobacco Smoking (ETS) (yes/no); (5) building age (before 1980, 1981–1990, 1991–2000, 2001–2005, after 2005); (7) air cleaning device (yes/no). To assess the potential for effect modification, we also examined models stratified by frequency of opening window in winter, building age and air cleaning device.

### Statistical methods

Univariate logistic regression models were used to obtain the association between each personal and environmental characteristic and SBS symptoms. Multiple logistic regression models were built to test the association between traffic exposure and general, mucosal and skin symptoms, controlling for other independent key covariates. Interaction terms were tested in multiple logistic regression models. Odds ratios with 95% confidence intervals (OR: 95% CI) were calculated. In statistical analysis, a two-tailed p-value below 0.05 was used to indicate statistical significance. The statistical analyses were conducted with SAS 9.3.

### Ethical issues

The Medical Research Ethics Committee of School of Public Health, Fudan University, approved both the study and the consent procedure.

## Results

There were 5487 questionnaires returned (65% response rate). 64 questionnaires answered by other persons (not parents or grandparents) were excluded in the analysis so that the gender and generation of the respondent is clearly classified. Table 1 details the prevalence and frequency (“Always (every week)”,”Sometimes” and “Never”) of SBS symptoms. Fatigue was the most prevalent symptom with 16.2% “Always” and 56.8% “Sometimes”.

**Table 1:** Prevalence of SBS symptoms (in the past three months).

	Male			Female		
	Always	Sometimes	Never	Always	Sometimes	Never
	N (%)	N (%)	N (%)	N (%)	N (%)	N (%)
Fatigue	427 (16)	1550 (57)	762 (28)	416 (17)	1402 (57)	642 (26)
Heavy-headed	105 (4)	880 (34)	1585 (62)	96 (4)	745 (32)	1459 (63)
Headache	77 (3)	1127 (43)	1416 (54)	108 (5)	984 (42)	1265 (54)
Nausea/dizziness	27 (1)	459 (18)	2057 (81)	37 (2)	407 (18)	1836 (81)
Difficulties concentrating	71 (3)	860 (33)	1663 (64)	92 (4)	747 (32)	1481 (64)
Itching eyes	92 (4)	712 (28)	1785 (69)	94 (4)	579 (25)	1651 (71)
Runny nose	89 (3)	958 (37)	1559 (60)	91 (4)	865 (37)	1374 (59)
Hoarse	141 (5)	1343 (51)	1146 (44)	136 6()	1177 (49)	1066 (45)
Cough	53 (2)	1349 (52)	1200 (46)	55 (2)	1182 (50)	1124 (48)
Dry facial skin	70 (3)	712 (28)	1801 (70)	74 (3)	598 (26)	1645 (71)
Scaling scalp or ears	84 (3)	620 (24)	1863 (73)	91 (4)	536 (23)	1690 (73)
Hands dry	25 (1)	274 (11)	2261 (88)	34 (1)	248 (11)	2029 (88)

Based on the score calculation, the percentages of cases with high general symptoms, mucosal symptoms and skin symptoms are 39.4%, 35.1% and 43.4%, respectively. The distribution of subject characteristics by exposure group is presented in Table 2. The participants were primarily women (77%) and parents (94%). More than half (64%) of participants owned their houses. The prevalence of ETS was high (51%). Overall, the distribution of personal and environmental characteristics for the two exposure categories were relatively close.

**Table 2:** Participants Personal and Environmental Characteristics Stratified by Traffic Exposure Category.

		Total (%)	Living near a highway (%)	Not living near a highway (%)
Gender	Female	3262 (77)	1281 (76)	1981 (77)
Generation	Parents	4019 (94)	1593 (94)	2426 (95)
Home ownership	Own	2796 (64)	1124 (65)	600 (63)
ETS	Yes	2227 (51)	896 (52)	1331 (50)
Frequency of opening window in winter	Always	278 (6)	105 (6)	175 (7)
	Sometimes	1176 (28)	453 (27)	723 (28)
	Never	1671 (66)	723 (67)	173 (65)
Building Age	After 2006	808 (18)	332 (19)	476 (18)
	2001–2005	1567 (36)	613 (35)	954 (36)
	1991–2000	1066 (24)	427 (25)	639 (24)
	1980–1990	620 (14)	245 (14)	375 (14)
	Before 1980	313 (7)	11 3(7)	200 (8)
Air cleaning device	Yes	477 (11)	172 (11)	305 (12)

The odds ratios for personal characteristics and environmental characteristics and SBS symptoms calculated in univariate logistic regression models are shown in Table 3. Significant variables identified from univariate logistic regression were consistent across general symptoms, mucosal symptoms and skin symptoms.

**Table 3:** ORs and 95% CIs from Univariate Logistic Models for SBS symptoms.

		OR (95%CI)		
		General Symptoms	Mucosal Symptoms	Skin Symptoms
Highway ^a^	Yes	**1.40 (1.24,1.59)**	**1.46 (1.29,1.66)**	**1.48 (1.31,1.68)**
Gender	Male	1	1	1
	Female	**1.44 (1.25,1.65)**	1.12 (0.97,1.29)	**1.38 (1.20,1.58)**
Generation	Parents	1	1	1
	Grandparents	**0.32 (0.24,0.43)**	**0.53 (0.40,0.71)**	**0.53 (0.40,0.69)**
Home ownership	Yes	**0.89 (0.80,1.04)**	0.90 (0.80,1.02)	**0.89 (0.79,1.00)**
ETS	Yes	**1.13 (1.02,1.25)**	1.07 (0.97,1.20)	**1.20 (1.07,1.33)**
Building age	After 2005	1	1	1
	2001–2005	**1.35 (1.15,1.58)**	**1.24 (1.06,1.47)**	**1.36 (1.16,1.60)**
	1991–2000	**1.34 (1.13,1.60)**	**1.22 (1.02,1.46)**	**1.37 (1.15,1.63)**
	1980–1990	1.18 (0.96,1.44)	**1.26 (1.02,1.55)**	**1.31 (1.07,1.60)**
	Before 1980	1.10 (0.85,1.41)	**1.34 (1.04,1.73)**	1.13 (0.87,1.45)
Window opening ^b^	Never	1	1	1
	Sometimes	**0.78 (0.68,0.89)**	**0.76 (0.67,0.88)**	**0.88 (0.77,1.00)**
	Always	**0.60 (0.47,0.77)**	**0.67 (0.52,0.86)**	0.80 (0.64,1.02)
Air cleaning device	Yes	**1.22 (1.02,1.46)**	**1.26 (1.05,1.51)**	1.07 (0.89,1.29)

a: Participants living near a highway or main road.

b: Frequency of window opening during winter.

Variables in bold are significant related to SBS symptoms with p-value less than 0.05.

Living near a main road or highway was a risk factor for each of the three groups of symptoms. The odds ratios for general symptoms, mucosal symptoms and skin symptoms were 1.40 (95% CI 1.24: 1.59), 1.46 (95% CI 1.29: 1.66) and 1.48 (95% CI 1.31:1.68), respectively. For personal characteristics, female gender was a risk factor for general symptoms and skin symptoms; grandparents’ generation was a protective factor for each of these three groups of symptoms; home ownership was a protective factor for general symptoms and skin symptoms. For environmental characteristics, ETS was a significant risk factor for general symptoms and skin symptoms; frequent (sometimes/always) window opening was a protective factor for each of these three symptoms; people living in a building constructed between 1981 and 2005 were at highest risk for each of the three symptom groups, using building constructed after 2005 as reference; using an air cleaning device was a risk factor for general symptoms and mucosal symptoms.

The adjusted odds ratios (aOR) for the association between living close to a highway and SBS symptoms remained significant in multivariable models. The aORs for association between living near a highway with general symptoms, mucosal symptoms and skin symptoms were 1.39, 1.48 and 1.42, respectively. Multivariable models are shown in Table 4.

**Table 4:** aORs and 95% CIs from Multivariable Model for SBS symptoms.

	OR (95%CI)		
	General Symptoms	Mucosal Symptoms	Skin Symptoms
**Primary Predictor**			
Near highway ^a^	**1.39 (1.21,1.59)**	**1.48 (1.29,1.70)**	**1.42 (1.24,1.63)**
**Covariates**			
Gender	**1.42 (1.21,1.67)**	1.17 (0.99,1.37)	**1.37 (1.16,1.61)**
Generation	**0.37 (0.25,0.54)**	**0.59 (0.41,0.84)**	**0.51 (0.36,0.72)**
Home ownership	**0.86 (0.74,0.99)**	0.92 (0.79,1.08)	**0.91 (0.78,1.06)**
ETS	**1.16 (1.01,1.32)**	1.04 (0.91,1.20)	**1.20 (1.05,1.37)**
Building age			
After 2005	Ref	-	-
Before 1980	1.12 (0.83,1.53)	**1.46 (1.07,1.99)**	1.30 (0.95,1.77)
1981–1990	1.14 (0.89,1.45)	**1.31 (1.02,1.68)**	**1.41 (1.11,1.81)**
1991–2000	**1.39 (1.13,1.72)**	**1.19 (0.95,1.48)**	**1.43 (1.16,1.77)**
2001–2005	**1.35 (1.11,1.63)**	**1.30 (1.07,1.59)**	**1.43 (1.18,1.74)**
Air cleaning device	**1.26 (1.02,1.55)**	1.13 (0.91,1.40)	1.00 (0.81,1.23)
Window opening			
Never	Ref	-	-
Always	0.82 (0.61,1.09)	0.77 (0.57,1.04)	0.86 (0.65,1.15)
Sometimes	**0.81 (0.70,0.95)**	**0.82 (0.70,0.96)**	0.93 (0.80,1.09)

a: living near a highway or main road.

Variables in bold are significant related to SBS symptoms with p-value less than 0.05.

The interaction terms between living near a highway or main road and the frequency of opening window in winter, building age and air cleaning device were tested in multivariable models for general, mucous and skin symptoms. However, no clear direction or significant findings were observed, so the interaction terms were not included in the final model.

## Discussion

Our study found that traffic exposure was a risk factor for increased odds of high SBS symptoms, with similar results across general symptoms, mucous symptoms and skin symptoms.

These findings are consistent with current literature that demonstrates an association between adverse health effects on the respiratory system and traffic exposure. A cross-sectional study of school-aged children in Taiyuan, China showed a positive relationship between ambient traffic pollutant concentrations and respiratory symptoms including wheezing and daytime or nocturnal breathlessness [23]. The Tasmanian Longitudinal Health Study similarly provides evidence of the adverse impact of traffic exposure on adult asthma [52]. A Californian study demonstrated an association between traffic related exposures including CO, NO_x_ and PM_2.5,_ especially in cool seasons, and asthma morbidity [53]. In 10 European cities, pollutants from busy roads are a major cause of chronic disease such as asthma [54]. An animal study with mice by Brandt et al. found diesel exhaust particle can cause severe asthma [55]. However, few studies have explored the association between traffic exposure and SBS symptoms. Wang et al. [3] performed a study in Chongqing and found SBS to be positively related to proximity to traffic, which supports our findings.

There are two hypothesized mechanisms for the development of adverse health effects caused by traffic, air pollution and noise [22, 32, 35, 56]. Traffic pollution is an important source of indoor air pollution, as demonstrated in an exposure assessment study [32]. The main pollutants generated by traffic include particulate matter less than 2.5 microns in diameter (PM 2.5), oxides of nitrogen (NO_x_) and sulfur dioxide (SO_2_). There is evidence that these pollutants are associated with cardiovascular mortality [56, 57]. Alternatively, noise from vehicles causes sleep and communication disturbances as well as hypertension and cardiovascular disease. A meta-analysis showed a significant positive association between noise annoyance and arterial hypertension [30].

Important covariates in our multivariable models included gender, age, home ownership, ETS, ventilation (frequency of window opening in the winter) and using an air cleaning device. Our study found that females experienced more symptoms than males, which is consistent with previous studies. Women have a more responsive immune system and are more prone to mucosal dryness and facial erythema [58]. Stenerg et al. [59] suggested that differences in working conditions and social status might explain why women report SBS more often than men. A study of 1000 random subjects also reported female gender as a main predictor of SBS symptoms [6].

In contrast to most studies, which reported no relationship with age, we found SBS symptoms to be less prevalent among older participants (grandparents). This may be attributed to the fact that our focus was residential SBS whereas previous studies have predominately studied office workers. Many of the older subjects are retired and subject to less psychosocial pressure [51].

This study also determined that people who own their apartments reported fewer SBS symptoms, perhaps reflecting their higher socioeconomic status [9].

ETS was shown to be a risk factor of SBS symptoms, agreeing with a study in Japan which found the odds ratio increases with hours of ETS exposure [8].

Low ventilation rate also increased the likelihood of high SBS prevalence, which may be attributed to an increase of stuffy odor and poor indoor air quality [14]. However, opening windows also increased the chance of traffic exposure. Since we do not have detailed information on how long and how much the windows were open, further analysis of the effects of window opening is limited. At present, we do not have a complete understanding of why utilizing an air cleaning device was a risk factor. It is possible that people with SBS symptoms are more likely to purchase an air cleaning device.

This study has some limitations. Highly detailed demographic information about participants was not available. Participants did not report variables such as age and education. Thus, we could only use surrogates such as caregiver generation (parent vs. grandparent) and home ownership (rent vs. own) to reflect the effects of age and socioeconomic status in our results [9].

Recall bias should be considered when both exposure and symptoms are collected in the same questionnaire. This could be considered a potential problem in our study, although the recall period we used was short (three months).

Our primary measure of traffic exposure was a self-reported variable based on distance to a “highway or main road” instead of a direct measurement of traffic exposure. While similar measures have shown this to be a good substitute for more detailed air pollution exposure assessments, there are limitations to this approach. Study participants may vary in their ability to estimate these distances, and since the outcome is binary, a dose-response analysis of the relationship between outcomes and distance from a highway is not possible. Geocoded household addresses combined with Beijing traffic databases would allow us to do a more objective assessment of traffic exposure.

Furthermore, as this is a cross-sectional study, we could not determine the causal relationship between exposures and health and could not eliminate the possibility that poor health conditions increased the likelihood of exposure [60].

## Conclusion

Our findings indicate an association between traffic related pollution and an adverse effect on symptoms generally associated with Sick Building Syndrome. This is among the first few studies that directly links traffic exposure to SBS symptoms in China. It will be important to replicate these results in other locations across China, especially as rapid urbanization increases exposure to traffic emissions across the country.

## Supporting Information

S1 File This is the data used for the analysis in this paper. (XLSX)

S2 File STROBE Checklist. (DOC)
