# Supplementary material for: Effect of Traffic Exposure on Sick Building Syndrome Symptoms among Parents/Grandparents of Preschool Children in Beijing, China
Source: PLoS One. 2015 Jun 18;10(6):e0128767. doi: 10.1371/journal.pone.0128767 (PMC4472806; doi:10.1371/journal.pone.0128767)
Supplement: S2 File — (DOC) [file pone.0128767.s002.doc]

STROBE Statement—checklist of items that should be included in reports of observational studies

|  | Item No | Recommendation | Page No. | Relevant text from manuscript |
| --- | --- | --- | --- | --- |
| **Title and abstract** | 1 | (*a*) Indicate the study’s design with a commonly used term in the title or the abstract | 1 | Self-administered questionnaires were distributed by teachers to legal guardians of children and then returned to teachers. |
|  |  | (*b*) Provide in the abstract an informative and balanced summary of what was done and what was found | 1 | This study found traffic exposure to be significantly associated with SBS symptoms. |
| Introduction | | |  |  |
| Background/rationale | 2 | Explain the scientific background and rationale for the investigation being reported | 2 | The rapid increase of SBS in China has become the focus of public health interest … |
| Objectives | 3 | State specific objectives, including any prespecified hypotheses | 4 | The aim of the paper is to study the association between traffic exposure and increased SBS symptoms controlling for other independent key household covariates. |
| Methods | | |  |  |
| Study design | 4 | Present key elements of study design early in the paper | 4 | Questionnaires were distributed by teachers to the legal guardians of children and then returned to teachers … |
| Setting | 5 | Describe the setting, locations, and relevant dates, including periods of recruitment, exposure, follow-up, and data collection | 4 | The China, Children, Homes and Health (CCHH) study is a cross-sectional study of children’s health in China … |
| Participants | 6 | (*a*) *Cohort study*—Give the eligibility criteria, and the sources and methods of selection of participants. Describe methods of follow-up  *Case-control study*—Give the eligibility criteria, and the sources and methods of case ascertainment and control selection. Give the rationale for the choice of cases and controls  *Cross-sectional study*—Give the eligibility criteria, and the sources and methods of selection of participants | 4 | From January through May, 2011, kindergartens in 11 districts in Beijing (Dongcheng, Xicheng, Chaoyang, Fengtai, Haidian and Shijingshan, Tongzhou, Changping and Daxing, Mentougou and Fangshan) were invited to participate in the study. |
|  |  | (*b*) *Cohort study*—For matched studies, give matching criteria and number of exposed and unexposed  *Case-control study*—For matched studies, give matching criteria and the number of controls per case |  |  |
| Variables | 7 | Clearly define all outcomes, exposures, predictors, potential confounders, and effect modifiers. Give diagnostic criteria, if applicable | 5 | Outcomes were assessed by asking: During the last three months, have you had any (or more) of the following symptoms… |
| Data sources/ measurement | 8* | For each variable of interest, give sources of data and details of methods of assessment (measurement). Describe comparability of assessment methods if there is more than one group | 6 | Living near a highway or main road was used as a metric for traffic exposure. Each participant was asked: “Is your home near a highway or main road within 200 meters (yes/no)?” |
| Bias | 9 | Describe any efforts to address potential sources of bias | 15 | … the recall period we used was short (3 months). |
| Study size | 10 | Explain how the study size was arrived at | 7 | There were 5487 questionnaires returned (65% response rate) … |
| Quantitative variables | 11 | Explain how quantitative variables were handled in the analyses. If applicable, describe which groupings were chosen and why |  |  |
| Statistical methods | 12 | (*a*) Describe all statistical methods, including those used to control for confounding | 6 | Univariate logistic regression models were used to obtain the association … |
|  |  | (*b*) Describe any methods used to examine subgroups and interactions | 13 | The interaction terms between living near a highway or main road and the frequency of opening window … |
|  |  | (*c*) Explain how missing data were addressed |  |  |
|  |  | (*d*) *Cohort study*—If applicable, explain how loss to follow-up was addressed  *Case-control study*—If applicable, explain how matching of cases and controls was addressed  *Cross-sectional study*—If applicable, describe analytical methods taking account of sampling strategy |  |  |
|  |  | (*e*) Describe any sensitivity analyses |  |  |

| Results | | |  |  |
| --- | --- | --- | --- | --- |
| Participants | 13* | (a) Report numbers of individuals at each stage of study—eg numbers potentially eligible, examined for eligibility, confirmed eligible, included in the study, completing follow-up, and analysed | 7 | There were 5487 questionnaires returned (65% response rate). 64 questionnaires answered by other person (not parents or grandparents) were excluded … |
|  |  | (b) Give reasons for non-participation at each stage |  |  |
|  |  | (c) Consider use of a flow diagram |  |  |
| Descriptive data | 14* | (a) Give characteristics of study participants (eg demographic, clinical, social) and information on exposures and potential confounders | 7-8 | Table 1 |
|  |  | (b) Indicate number of participants with missing data for each variable of interest |  |  |
|  |  | (c) *Cohort study*—Summarise follow-up time (eg, average and total amount) |  |  |
| Outcome data | 15* | *Cohort study*—Report numbers of outcome events or summary measures over time |  |  |
|  |  | *Case-control study—*Report numbers in each exposure category, or summary measures of exposure |  |  |
|  |  | *Cross-sectional study—*Report numbers of outcome events or summary measures | 9 | Table 2 |
| Main results | 16 | (*a*) Give unadjusted estimates and, if applicable, confounder-adjusted estimates and their precision (eg, 95% confidence interval). Make clear which confounders were adjusted for and why they were included | 10-12 | Table 3 & 4 |
|  |  | (*b*) Report category boundaries when continuous variables were categorized |  |  |
|  |  | (*c*) If relevant, consider translating estimates of relative risk into absolute risk for a meaningful time period |  |  |
| Other analyses | 17 | Report other analyses done—eg analyses of subgroups and interactions, and sensitivity analyses | 13 | The interaction terms between living near a highway or main road and the frequency of opening window … |
| Discussion | | |  |  |
| Key results | 18 | Summarise key results with reference to study objectives | 13 | Our study found that traffic exposure was a risk factor for increased odds of high SBS symptoms … |
| Limitations | 19 | Discuss limitations of the study, taking into account sources of potential bias or imprecision. Discuss both direction and magnitude of any potential bias | 15 | This study has some limitations … |
| Interpretation | 20 | Give a cautious overall interpretation of results considering objectives, limitations, multiplicity of analyses, results from similar studies, and other relevant evidence | 13-15 | These findings are consistent with current literature that demonstrates … |
| Generalisability | 21 | Discuss the generalizability (external validity) of the study results | 16 | It will be important to replicate these results in other locations in China … |
| Other information | | |  |  |
| Funding | 22 | Give the source of funding and the role of the funders for the present study and, if applicable, for the original study on which the present article is based |  | Natural Science Foundation of China (51136002) |

*Give information separately for cases and controls in case-control studies and, if applicable, for exposed and unexposed groups in cohort and cross-sectional studies.

**Note:** An Explanation and Elaboration article discusses each checklist item and gives methodological background and published examples of transparent reporting. The STROBE checklist is best used in conjunction with this article (freely available on the Web sites of PLoS Medicine at http://www.plosmedicine.org/, Annals of Internal Medicine at http://www.annals.org/, and Epidemiology at http://www.epidem.com/). Information on the STROBE Initiative is available at www.strobe-statement.org.
